# Genetic features of *Mycobacterium tuberculosis* modern Beijing sublineage

**DOI:** 10.1038/emi.2016.14

**Published:** 2016-02-24

**Authors:** Qingyun Liu, Tao Luo, Xinran Dong, Gang Sun, Zhu Liu, Mingyun Gan, Jie Wu, Xin Shen, Qian Gao

**Affiliations:** 1 Key Laboratory of Medical Molecular Virology of Ministries of Education and Health, Institutes of Biomedical Sciences and Institute of Medical Microbiology, School of Basic Medical Sciences, Fudan University, Shanghai 200032, China; 2 Laboratory of Infection and Immunity, School of Basic Medical Science, West China Center of Medical Sciences, Sichuan University, Chengdu, Sichuan 610041, China; 3 Institute of Biostatistics, School of Life Sciences, Fudan University, Shanghai 200433, China; 4 Department of TB Control, Shanghai Municipal Centers for Disease Control and Prevention, Shanghai 200336, China

**Keywords:** ancient sublineage, Beijing family, microevolution, modern sublineage, *Mycobacterium tuberculosis*

## Abstract

*Mycobacterium tuberculosis* (MTB) Beijing strains have caused a great concern because of their rapid emergence and increasing prevalence in worldwide regions. Great efforts have been made to investigate the pathogenic characteristics of Beijing strains such as hypervirulence, drug resistance and favoring transmission. Phylogenetically, MTB Beijing family was divided into modern and ancient sublineages. Modern Beijing strains displayed enhanced virulence and higher prevalence when compared with ancient Beijing strains, but the genetic basis for this difference remains unclear. In this study, by analyzing previously published sequencing data of 1082 MTB Beijing isolates, we determined the genetic changes that were commonly present in modern Beijing strains but absent in ancient Beijing strains. These changes include 44 single-nucleotide polymorphisms (SNPs) and two short genomic deletions. Through bioinformatics analysis, we demonstrated that these genetic changes had high probability of functional effects. For example, 4 genes were frameshifted due to premature stop mutation or genomic deletions, 19 nonsynonymous SNPs located in conservative codons, and there is a significant enrichment in regulatory network for all nonsynonymous mutations. Besides, three SNPs located in promoter regions were verified to alter downstream gene expressions. Our study precisely defined the genetic features of modern Beijing strains and provided interesting clues for future researches to elucidate the mechanisms that underlie this sublineage's successful expansion. These findings from the analysis of the modern Beijing sublineage could provide us a model to understand the dynamics of pathogenicity of MTB.

## INTRODUCTION

*Mycobacterium tuberculosis* (MTB) isolates are currently classified into seven major lineages (lineage 1–7), and among them, lineage 2 is one of the most successful lineages with increasing prevalence in global population.^1^ Lineage 2, also known as Beijing family, was first found to be dominant in South and East Asia.^2,3^ In the past two decades, Beijing strains caused a great concern because of the frequent associations with higher mutation rate, hypervirulence, immune evasion, treatment failures and drug resistance.^4^ However, these associations or characteristics varied among different studies and were inconclusive, suggesting that genetic heterogeneity might exist within this family.^4,5,6,7^

Initially, Beijing strains were found highly homogenous based on genotyping data.^8^ Study from Dick van Soolingen *et al.* suggested that they were recently selected by Bacille Calmette-Guerin (BCG) vaccination,^8^ but recent studies found that the diversity within Beijing strains is higher than previously estimated and the expansion of Beijing strains is prior to the BCG vaccination.^1,9,10^ Based on the existence or absence of IS6110 insertion(s) in the noise transfer function (NTF) region, Beijing family trains were further divided into modern (typical) and ancient (atypical) Beijing sublineages.^11^ Of them, modern Beijing sublineage is the most prevalent Beijing sublineage in worldwide regions except in Japan and Korea.^2,3,5,11^ However, even in Japan, a rapid emergence and increasing prevalence of modern Beijing sublineage strains were reported.^12^ The increasing prevalence of modern Beijing strains suggests that this sublineage might exhibit selective advantage over ancient Beijing sublineage. This selective advantage could be evaluated through various virulence-associated characters. For example, modern Beijing strains induced a lower level of proinflammatory cytokines (IL-1β, IFN-γ, IL-22) compared with ancient Beijing strains in peripheral blood mononuclear cells.^13^ In both mice pulmonary infection and macrophage infection models, modern Beijing strains were more likely to exhibit highly virulent phenotypes than ancient Beijing strains.^14^

Although modern Beijing strains have been a research hotspot in recent years, the genetic basis that contributes to their global prevalence is still unclear. As MTB lack horizontal gene transfer and recombination, the genomic evolution of MTB was characterized by stepwise accumulation of mutations.^15,16^ Identification of modern Beijing-specific genetic changes may provide important clues for understanding their phenotypic advantages. Modern Beijing strains are known to display missense alterations in three putative mutation repair genes (*Rv3908*, *mutT2* and *ogt*) and these mutations were supposed to confer advantages of the rapid adaption to new environment as they might increase mutation rate.^6,9^ However, it is still not clear whether they play a role in the adaptation of modern Beijing strains as the influence of these mutations have not been validated yet.^4,17^ Thus, a comprehensive characterization of genetic features of modern Beijing strains is still needed. Previously, Anita C. Schurch *et al* have defined a minimal set of polymorphisms (51 single-nucleotide polymorphisms (SNPs)) for modern Beijing strains through typing 150 MTB strains with a subset of the SNPs they identified, and concluded that mutations in the regulatory network underlay its recent clonal expansion.^18^ More recently, Matthias Merker *et al.* examined the characteristics of modern Beijing strains as 81 SNPs based on whole-genome sequencing of 110 globally collected MTB Beijing isolates.^19^ The inconsistency of the two studies indicated that the variation was due to different sample sets they studied. Thus, investigating more representative isolates would lead to more accurate definition of modern Beijing genetic features. In recent years, a large number of whole-genome sequencing data of MTB Beijing strains has been published,^1,10,19,20,21^ which provided the possibility to further illustrate evolutionary route of modern Beijing sublineage. In this study, taking advantage of large number of online available sequencing data of MTB Beijing strains, we defined the genetic features of modern Beijing sublineage more precisely and further demonstrated that these changes would probably cause functional effects.

## MATERIALS AND METHODS

### Genome sequencing data and SNPs/INDELs calling

Whole-genome sequencing data of MTB isolates were obtained from National Center for Biotechnology Information (NCBI) and European Nucleotide Archive (ENA. We used both SNP G-A in codon 176 of *Rv2952* and SNP C→G in codon 20 of *Rv2450c* to identify Beijing strains as previously described.^5^ Totally, whole-genome sequencing data of 1082 Beijing strains were included in our study and listed in Supplementary Table S1. The strains were from 15 countries with the major contribution from China and Russia (Supplementary Table S1). There were 193 strains from China, where Beijing strains originated and the highest genetic diversity was observed. Thus, we argue that the data set included in our study in geographically and genetically representative. The FASTQ format raw sequencing dat were processed by Scythe (https://github.com/ucdavis-bioinformatics/scythe) and Sickle (https://github.com/najoshi/sickle) for trimming adapter and low-quality bases, respectively. We discarded low-quality bases with Phred quality scores lower than 20. After mapping to the reference genome MTB H37Rv (GenBank accession NC_000962.3) with Burrows–Wheeler Aligner,^22^ SNPs were called against the reference with a minimum depth of 20 folds coverage using SAMtools.^23^ For insertions and deletions (INDELs) calling, we used Velvet^24^ for *de novo* assembly and used BLAT^25^ to align assembled contigs with the reference genome MTB H37Rv. The INDELs between each sequenced isolate and the reference genome were then identified from the alignment file. Mobile genetic elements, repetitive sequences, PE/PPE and PE-PGRS gene families that might cause incorrect read alignment were excluded in this study.

### Identify modern Beijing genetic features

A maximum likelihood (ML) tree was constructed in MEGA5^26^ using the default parameters with the union of 55630 SNPs identified in the sequenced data and the robustness of the ML tree was validated by a 1000-time bootstrap test. Another four published Beijing strains genome data (CCDC5079, accession: NC_017523; CCDC5180, accession: NC_017522.1; X122, accession: CM001044; HN878, accession: CM001043; MTB 210, accession: ADAB00000000) were also included for a comprehensive view of Beijing family phylogenetic structure. The phylogenetic tree was visualized with Fig Tree (http://tree.bio.ed.ac.uk/software/figtree/). We applied two approaches to identify modern Beijing-specific mutations. First, we identified modern Beijing strains based on *mutT2 G58R*, *ogt* codon 12 (GGG-GGA) mutations and IS6110 insertion(s) in NTF region. Then we wrote a Perl script to identify the SNPs and INDELs that were uniformly presented in all modern Beijing strains but absent in closest ancient Beijing strains. Second, we reconstructed the ML phylogeny of Beijing strains based on the SNPs called from all strains in this study and further used MEGA5 to reconstruct the most recent common ancestor (MRCA) sequence of all modern Beijing strains (MRCA-modern) and also the MRCA sequence of the closest ancient Beijing node (MRCA-ancient. Through blast the two MRCA sequences, we identified modern Beijing-specific SNPs as the SNPs presented in MRCA-modern sequence against MRCA-ancient sequence.

### Bioinformatics analysis

We downloaded gene annotation file from TBDB (http://www.tbdb.org/) and identified synonymous and nonsynonymous mutations based on the reference genome H37Rv. We download protein sequence from Uniprot and NCBI by choosing ‘Mycobacteria' branch and perform PSI-BLAST^27^ for each TB protein-coding gene. Then, we could get conservation score for each position of each TB gene, with score ranges from 0 to 4.36 with higher score indicating higher conservation. If the score is higher than 2.5, the corresponding site can be defined as a conservative site. For all nonsynonymous SNPs, we could detect whether it is located at a conservative position. We used SMART (http://smart.embl-heidelberg.de/) to identify protein domains and analyze the domain architectures. We performed functional enrichment analysis for the genes influenced by the modern Beijing nonsynonymous SNPs using annotation system from Tuberculist,^28^ GO,^29^ KEGG^30^ and COG.^31^ For each annotated gene set, we performed Fisher's exact test to find out ones that were significantly over-represented in the genes with modern Beijing SNPs. The ChIP-Seq data sets were downloaded from TBDB. We used MEME^32^ to predict PWM for each transcription factor (TF) and scan the whole genome to get potential TF binding site (TFBS). Then, we scanned the input SNP to find whether it located at TFBS and changed the binding potential of the corresponding TF. All SNPs were mapped to the TFBS at each position, and the TF binding potential score was calculated for the original TFBS and altered TFBS.

### Luciferase report system assays

For the seven genes that were predicted to be affected by the mutations in TFBS, about 500 bp upstream sequences from start codon of these genes were amplified by PCR from both ancient Beijing (wild-type) and modern Beijing (mutant-type) strains, and then cloned into the pSMT3L-EGFP vector. A total of 14 plasmids were obtained and transferred into *Mycobacterium smegmatis* through electroporation. The *M. smegmatis* strains carrying these plasmids were cultured in 7H9 medium. Cultures (10 mL) of *M. smegmatis* were grown to OD_600_ = 0.6∼0.8 and then standardized to OD_600_ = 0.1. Luciferase activity was determined immediately after the collection of the cultures through the GloMax^®^ 20/20 Luminometer (Promega). Three biological repeats were performed.

### Ethic statement

All the whole-genome sequencing data analyzed were downloaded from online available resource (NCBI and ENA) and we did not generate new sequencing data in this study. Thus, there is no ethic/consent statement to make.

## RESULTS

### Genetic features of modern Beijing sublineage

**Figure 1:**
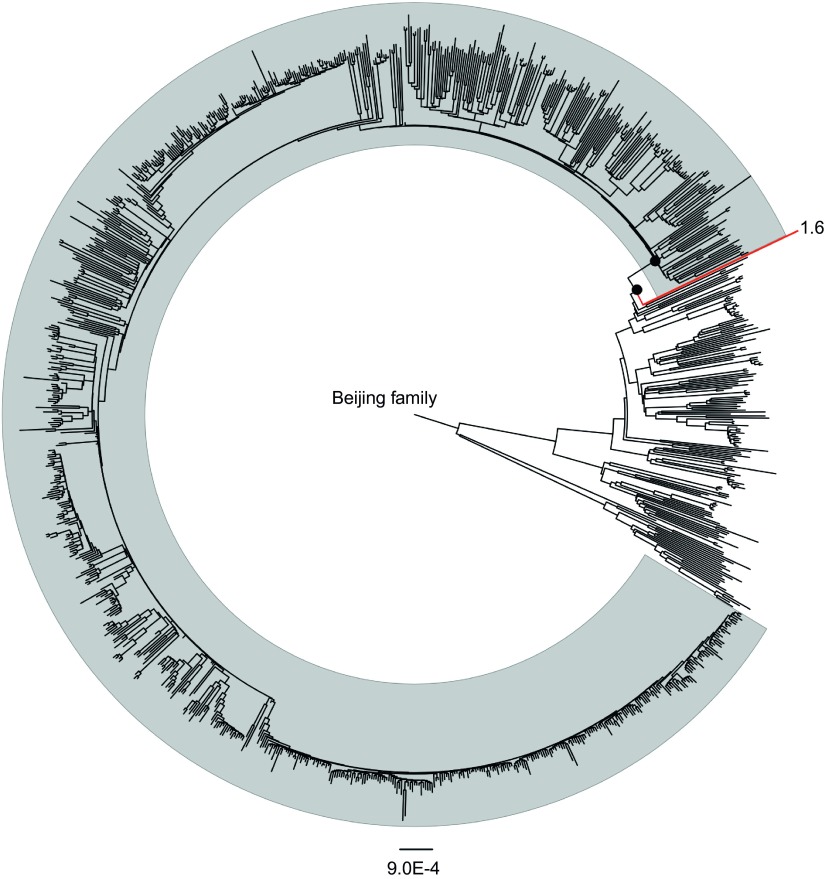
Maximum-likelihood phylogeny of 1082 MTB Beijing isolates. Strains shown in gray shadow represent modern Beijing sublineage while others belong to ancient Beijing sublineage. The two dark circles represent the MRCA sequences of modern Beijing strains and the closest ancient branch, respectively. Ancient Beijing strain 1-6 that harbored mutT2 G58R mutation but not IS6110 insertion in NTF region is marked in the phylogeny.

Whole-genome sequencing data of 1082 MTB Beijing isolates representing global diversity were downloaded from NCBI and ENA. Through mapping the sequencing reads to reference genome H37Rv, we called SNPs of each isolate against the H37Rv reference genome sequence. Among the 1082 Beijing isolates, 900 were determined to belong to modern Beijing sublineage by matching all three genetic markers (*mutT2* G58R, *ogt* codon 12 GGG-GGA and IS6110 insertion in NTF region), and 181 were determined as ancient Beijing isolates in the absence of these genetic markers. One isolate named ‘1-6' (sampled from Shanghai, China according to the original study) showed ambiguous characteristics according to the definitions. This isolate, although carrying *mutT2* G58R mutation, was detected as wild type in *ogt* codon 12 and showed intact NTF region without IS6110 insertion. We reconstructed a maximum-likelihood phylogeny of the 1082 MTB Beijing isolates (Figure 1). We applied two approaches to define the genetic features of modern Beijing sublineage. First, we identified the SNPs and insertions/deletions that were uniformly presented in modern Beijing strains but absent in the closest ancient Beijing branches. Second, we identified modern Beijing-specific SNPs through reconstructing the MRCA sequence of modern Beijing sublineage and compared it with the MRCA sequence of the closest ancient Beijing node (Figure 1). Totally, 44 SNPs and 2 short genomic deletions were identified as modern Beijing-specific genetic features. Compared with the results from Matthias Merker *et al.,*^19^ we excluded 43 SNPs that were not specific to modern Beijing strains but newly identified six SNPs and one genomic deletion. Comparing our results with the previous study by Schurch AC *et al.,*^18^ we narrowed down the minimum set by excluding seven mutations and newly identified two short genomic deletions. The reason we narrowed down the minimum SNP set is that we included more ancient Beijing strains that were genetically closer to modern Beijing strains. The major contribution to the reduction of minimum SNP set was from the strain ‘1-6.' This isolate showed closest distance to modern Beijing sublineage but was excluded from the expanded branch of modern Beijing sublineage (Figure 1). The isolate ‘1-6' harbored *mutT2* G58R mutation but not *ogt* codon 12 mutation or IS6110 insertion in NTF region. Therefore, the mutations present in strain ‘1-6' should not be modern Beijing-specific mutations and *mutT2* G58R mutation was actually not strictly restricted to modern Beijing sublineage.

The 44 modern Beijing SNPs consist of 27 nonsynonymous (including one premature stop codon mutation), 13 synonymous and four intergenic mutations (Supplementary Table S2). The premature stop codon mutation in Rv2180c caused the coding protein truncated from 295 to 249 amino acids (Table 1). For the two short deletions, one is a single-nucleotide deletion in the overlap region of genes Rv2147c and Rv2148c that caused frameshift mutations for both genes. Rv2147c was shortened from 241 to 218 amino acids and Rv2148c had a frame shift alteration of the 5 tail amino acids (Table 1). The other was a 19-nucleotide deletion in gene Rv1730c, leading to the premature termination of Rv1730c with the coding protein length shortened from 517 to 418 amino acids, which likely results in inactivation of Rv1730c (Table 1). Among the four genes above, Rv1730c and Rv2147c were essential genes.^33^ Rv1730c possibly codes a penicillin-binding protein, was involved in cell wall biosynthesis and may also act as a sensor of external penicillin.^33^ Rv2147c was a core mycobacterial gene that is specific for mycobacteria and intracellular mycobacterial pathogens.^34^ It is noteworthy that all the four genes mentioned above were located in cell wall or cell membrane. As for bacteria pathogens, the proteins in cell envelope are crucial to maintain the stability and integrity of cell and play an important role in virulence, host cell interaction and immune responses. Thus, these changes might have functional impacts on the cell envelope structure or host immunological recognition.

### Potential functional changes revealed by bioinformatics analysis

Of the 27 genes carrying nonsynonymous mutations, we further performed conservative analysis and gene function enrichment analysis to predict their functional effects. Totally, 10 genes with nonsynonymous mutations encoded proteins located in cell wall or outer membrane (Supplementary Table S2), and these mutated genes were prone to frequent contact with host immune system. Conservative analysis showed that 18 of the 27 nonsynonymous SNPs were in the comparative conservative codons (Supplementary Table S3), suggesting that they might lead to functional changes of the related proteins. Among the genes with mutations in conservative codons, nine encoded enzymes and involved in metabolism or signal transduction (Supplementary Table S3). Gene function enrichment analysis revealed that the genes with nonsynonymous mutations were mainly enriched in regulatory protein network (Table 2, *P = 0.029*), which was also revealed by Schurch AC *et al.* earlier.^18^ In modern Beijing strains, PknA has two adjacent amino changes (Q369R and Q370P) in the low-complexity region of outer membrane domain and both changes altered the property of the amino acids. Among these affected regulatory proteins, PknA is widely used to transduce extracellular signals into appropriate intracellular responses and involved in numerous cellular processes, and the alteration of sensor domain might lead to a difference in response to external stimulus.^35,36^ *Rv0890c* and *Rv2488c* are both LuxR family regulators involved in MTB dormancy.^37,38^ Previous study showed that the loss of a LuxR family regulator *Rv0386* (an adenylate cyclase) decreased immunopathology in animal tissues and bacterial survival.^39^ Coding changes in transcriptional regulators are considered to be rare because their multifunctional roles and a small change would produce widespread detrimental effects, and mutations in regulatory networks were also thought to involve in bacteria's adaptation to host.^40^ Thus, the significant enrichment of regulatory network proteins might be a reflection of modern Beijing sublineage's adaptive microevolution.

### Modern Beijing SNPs altered downstream genes expression

**Figure 2:**
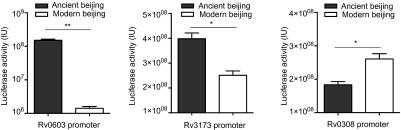
Modern Beijing-specific SNPs alter the expression level of downstream genes. The promoters with modern Beijing-specific mutations have been tested for transcriptional activity against promoters without respective mutations through luciferase report system in *M. smegmatis*. Data presented are mean values from three independent experiments. Error bars, 95% confidence intervals.

Among the 44 modern Beijing-specific SNPs, five were predicted to locate in the TFBSs and might alter the expression of downstream genes (Supplementary Table S4). We further experimentally investigated whether these SNPs would affect downstream gene expression through luciferase report system in *M. smegmatis*. Three SNPs were verified to have impacts on the expression of downstream genes (Figure 2). Among them, mutation 16 A-C in the promoter of *Rv0603* caused ∼100-fold downregulation. *Rv0603* possibly codes an exported protein but its function was unknown.^41,42^ Thus, it is hard to infer the biological changes due to low expression of *Rv0603*. Mutation in predicted promoter of *Rv3173c* led to 1.4-fold decreased regulation. However, this small change could amplify into great influence as *Rv3173c* codes a TetR/AcrR family transcriptional regulator that regulates a large number of genes.^43^ *Rv0308* probably codes a conserved integral membrane protein and mutation in the predicted promoter of *Rv0308* led to ∼1.6-fold increase of expression. The other two mutations did not have significant impact on the downstream genes' expression. These results confirmed the functional effects of modern Beijing SNPs, but due to the poor knowledge of these genes' functions, we could not make an inference for the biological effects of these alterations. Thus, it will be interesting to verify whether these alterations would contribute to some phenotype changes. The other three SNPs that did not change the downstream genes' expression are shown in Supplementary Figure S1.

## DISCUSSION

A recent study based on phylogeographic and coalescent analyses of large number of Beijing strains indicated that Beijing family emerged around 30 000 years ago in southern East Asia, which was accompanied with the early colonization by modern humans in this area.^10^ After that, Beijing strains branched into several major sublineages during the coevolution with human beings in Asia. Among them, modern Beijing sublineage was the latest branch but experienced the most significant expansion during the neolithic demographic transition (NDT, 6000–7000 years ago), suggesting that this sublineage had successfully adapted to the environment change of increasing human population densities during NDT.^10^ Thus, identification of genetic difference between modern Beijing and other Beijing sublineages would help us understand the pathogenicity evolution of MTB. In this study, through analyzing large number of published whole-genome sequencing data of MTB, we reconstructed the most representative phylogeny of Beijing strains and characterized the genetic features that were restricted to modern Beijing sublineage. We argue that the narrowing down of modern Beijing-specific SNPs in our study compared with previous studies is more precise because we included a lot more representative Beijing strains for analysis.

We performed several analysis or experiments to investigate the functional effects of modern Beijing genetic features. Through bioinformatics analysis, we found that three genes were truncated and one gene was frameshifted. Truncations of genes usually lead to loss of gene function, but due to the poor annotation of these genes, we could not speculate the biological influence directly. Yet, all the four genes were located in cell membrane or cell wall, genes of which category are frequently associated with maintaining cell envelop integrity or immunological recognition. Thus, revealing these genes' function would help us understand the microevolution of MTB modern Beijing strains. Nonsynonymous mutations in conservative codons might lead to changes in the enzyme activity or structure of encoded proteins. Our conservative analysis of the 27 nonsynonymous mutations demonstrated that 18 of them were located in conservative sites, which further supported the functional effects of modern Beijing genetic features. Remodeling of global regulatory networks is a key route for bacterial pathogen adaptation to the fluctuations of host environment;^44^ thus, the significant enrichment in regulatory protein category of modern Beijing-specific nonsynonymous mutations might be an indicator of pathogen microevolution.

Besides, several other mutations that could affect the virulence of modern Beijing strains are also listed. Modern Beijing strains have a nonsynonymous mutation at a conservative site in *secA2*. MTB has two SecA proteins, SecA1 and SecA2; SecA1 handles ‘housekeeping' export while SecA2 exports a specific subset of virulence factors and is necessary to elude the oxidative attack of macrophages.^45,46^ Another conservative-site mutation was observed in *sseA*, which codes a putative thiosulfate sulfurtransferase, and a study showed that mutation in this gene could lead to enhanced growth in macrophages relative to *in vitro* growth.^47^ More recently, de Keijzer J *et al.* further found that SseA was among the four proteins that were differentially regulated between ancient and modern Beijing sublineages.^48^ Mutation in *glcB* was at a highly conservative site and this gene encodes a malate synthase. GlcB takes part in the glyoxylate shunt and has been implicated as a virulence factor, which was also important for MTB survival under adverse conditions, such as low oxygen, nonreplicative states and the intracellular environment.^49^ Thus, it will be interesting to verify whether these mutations would cause some changes on protein functions.

However, our study still has several limitations. First, due to the lower prevalence of ancient Beijing strains, the number of isolates analyzed belonging to the modern sublineage is much higher than those from the ancient Beijing sublineage. Thus, if more ancient Beijing strains that showed even closer relationship to modern Beijing sublineage were sequenced, the minimum set of modern Beijing-specific genetic features might be further reduced. Second, the approach we used to validate the change of downstream genes' expression was through luciferase report system in *M. smegmatis.* Although this approach was commonly accepted and used for this purpose, we failed to further confirm these findings in MTB isolates due to the requirement for biosafety level 3 lab to culture MTB and extract RNA.

In conclusion, we identified the genetic features that were restricted to modern Beijing sublineage and revealed that they had functional effects. Through characterizing the modern Beijing-specific genetic changes, our findings provided new clues to elucidate the successful expansion or hypervirulence of MTB modern Beijing strains. As Beijing family is one of the most successful lineages of global MTB strains, these investigations into modern Beijing sublineage could provide us a model to understand the dynamics of pathogenicity in MTB.

## Figures and Tables

**Table 1:** Genes with significant alterations in modern Beijing strains

**Gene name**	**Genetic changes**	**Alteration**	**Cellular localization**	**Description**
*Rv1730c*	19 bp deletion from codon 438 to 444	517–>418 amino acids	Cell wall and cell processes	Essential, possible penicillin-binding protein, involved in cell wall biosynthesis and may also act as a sensor of external penicillin
*Rv2147c*	1 bp deletion in start codon	241–>218 amino acids	Cell membrane	Essential, a core mycobacterial gene, conserved in mycobacterial strains
*Rv2148c*	1 bp deletion in codon 258	Frameshift of 5 amino acids	Cell membrane	Nonessential, conserved protein, function unknown
*Rv2180c*	A premature stop codon mutation	295–>249 amino acids	Cell wall and cell processes	Nonessential, probable conserved integral membrane protein, function unknown

**Table 2:** Modern Beijing-specific mutations that are enriched in the gene category of regulatory network and other mutations with potential functional effects

**Categories**	**Gene**	**Codon change**	**Gene description**
**Regulatory network**	*pknA*	Q370P	Transmembrane serine/threonine protein kinase A
		Q369R	Transmembrane serine/threonine protein kinase A
	*Rv0452*	H125D	Possible transcriptional regulatory protein
	*Rv0890c*	E234G	Probable LuxR family transcriptional regulatory protein
	*Rv2488c*	T265I	Transcriptional regulator, LuxR family
	*Rv3173c*	Promoter mutation	TetR/AcrR family transcriptional regulator
**Other categories**	*secA2*	V410M	Possible preprotein translocase ATPase SecA2
	*sseA*	E276K	Probable short-chain dehydrogenase/reductase
	*glcB*	G104S	Probable malate synthase G GlcB
