# Supplementary material for: Genetic features of Mycobacterium tuberculosis modern Beijing sublineage
Source: Emerg Microbes Infect. 2016 Feb 24;5(2):e14–. doi: 10.1038/emi.2016.14 (PMC4777927; doi:10.1038/emi.2016.14)

Supplementary Figure S1 The other three SNPs that didn't change expression of downstream genes. Modern Beijing SNPs in the predicted promoters of tcrA, Rv0238 and phhB didn’t change the down stream genes expressions.

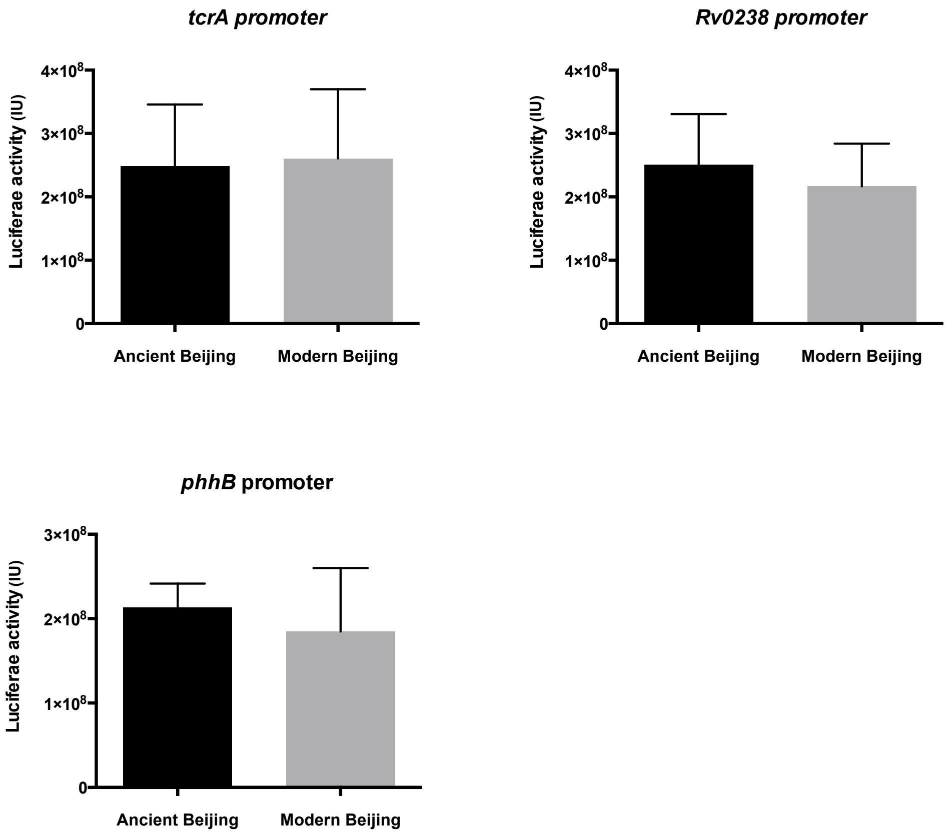

Supplement: Supplementary Information [file emi201614x5.pdf]
